# CD20/CD79a/PAX5/CD3-negative post-transplant lymphoma with aberrant actin and desmin co-expression—a potential differential diagnostic pitfall between PTLD and PTSMT

**DOI:** 10.1007/s00277-021-04394-2

**Published:** 2022-02-18

**Authors:** Jan-Theile Suhren, Jerome Schlué, Hans Kreipe, Kais Hussein

**Affiliations:** Institute of Pathology, Medizinische Hochschule Hannover, Carl-Neuberg-Strasse 1, 30625 Hannover, Germany

Dear Editor,

Post-transplant lymphoproliferative disorders (PTLD) are commonly CD20^+^ and rarely CD20^−^ [1]. Co-expression of mesenchymal/muscle markers is unusual in PTLD and non-PTLD lymphomas [2]. The main clinical differential diagnosis is the manifestation of a post-transplant smooth muscle tumour (PTSMT) [3, 4]. Both types of post-transplant tumours can manifest at any anatomic location and present with similar clinical features [3, 4].

**Fig. 1:**
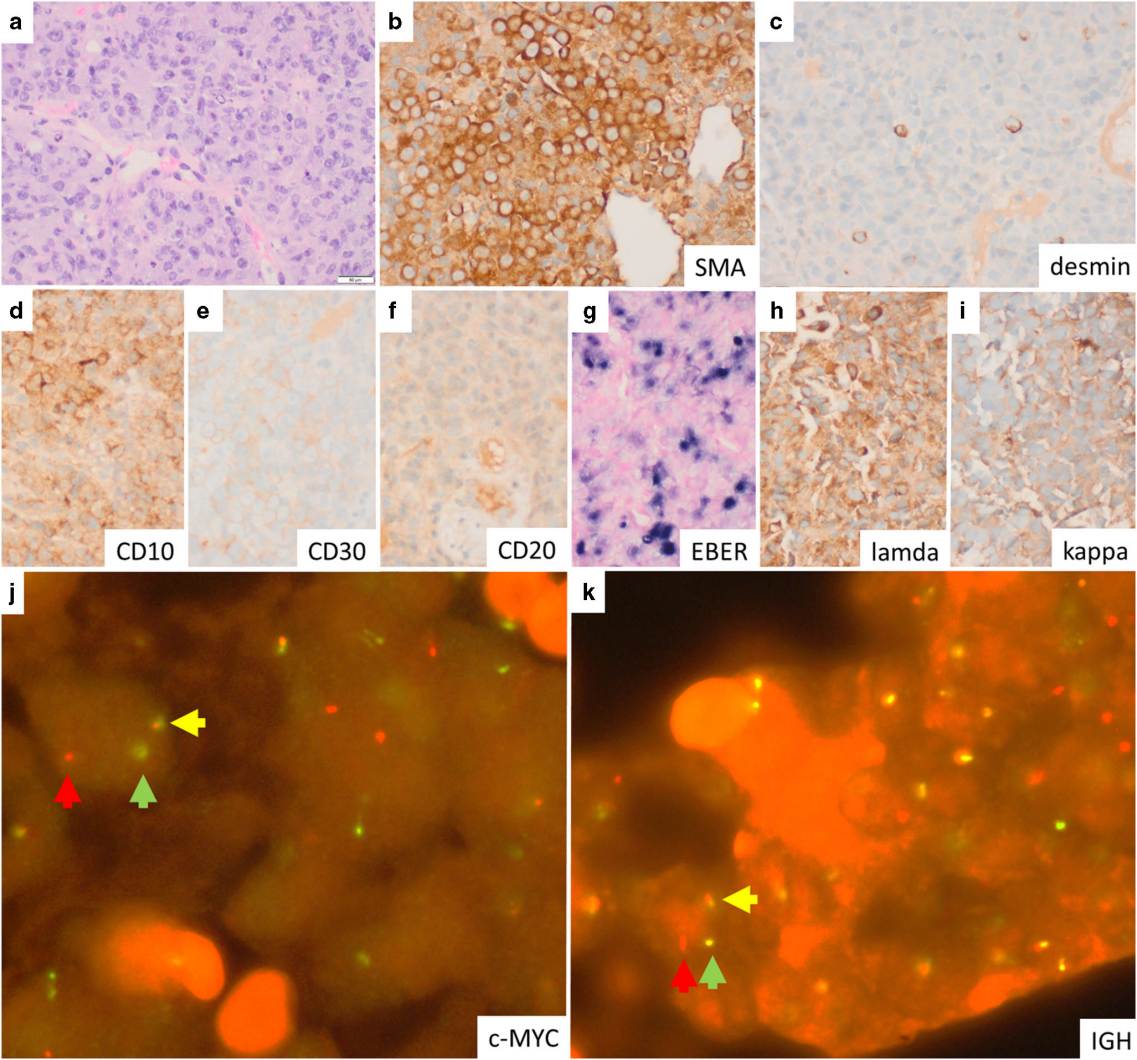
(**a**) Histology of the biopsy showed large round to oval blastoid cells with large nuclei with prominent nucleoli (haematoxylin eosin stain). (**b**–**i**) Immunohistochemistry revealed lambda-positive plasmablastic tumour cells with aberrant mesenchymal marker expression (smooth muscle actin/SMA > 90% of tumour cells; < 5% desmin). Positivity was detected also for EBER (~ 50%), c-MYC (> 90%), CD10 (> 90%), CD30 (30–40%; weak), Ki67 (> 90%) and PDL1 (~ 60%); c-MYC, Ki67 and PDL1 are not depicted. The following markers were negative: caldesmon, CD20, CD79a, PAX5, CD3, BCL2, granzyme B, perforin, CD52, CD34, CD117 and CK5/14. (**j**, **k**) Fluorescence in situ hybridisation showed split signals of c-MYC and IGH. BCL2 and BCL6 showed normal configuration (not depicted). Lambda light chain restriction was confirmed by RT-PCR; PCR showed a monoclonal IGH signal, and short tandem repeat PCR indicated recipient origin of the tumour cells (not depicted). Original magnification: **a**–**i** each × 400; **j** and **k** each × 1000

In our case study, the male patient had previously been diagnosed with primary sclerosing cholangitis, autoimmune hepatitis and colitis ulcerosa. Seventy-four months after liver transplantation, at the age of 22 years, the patient complained about upper abdominal pain and fever. Subsequently, a left-sided liver tumour of 4.4 cm was found. Biopsy showed a large cell neoplasm, which was CD20^−^/CD79a^−^/PAX5^−^/CD3^−^ but diffusely smooth muscle actin^+^, focal desmin^+^, partial Epstein-Barr virus (EBV)**-**encoded small RNAs (EBER)^+^ as well as CD10^+^, CD30^(+)^ and MYC proto-oncogene (c-MYC)^+^ (Fig. 1). At first, an unusual high-grade PTSMT was suspected. However, further analyses revealed negativity for caldesmon but lambda light chain restriction and rearrangements of c-MYC and immunoglobulin heavy chain (IGH). Therefore, the diagnosis was plasmablastic PTLD and not PTSMT.

The patient refused treatment at the local hospital and died 5 months later.

Typically, sarcomatoid dedifferentiated lymphomas show spindle cells with high-grade atypia [5]. In contrast, PTSMT usually show spindle cells with low-grade atypia; however, high-grade PTSMT has been reported in some patients [4]. EBV-association and c-MYC expression can be found in both PTLD and PTSMT, while CD10 and CD30 are usually negative in PTSMT [3]. In most cases, EBV can be detected in all tumour cells, but, as in our case, partial EBER positivity could be the result of partial genetic loss of EBV in a sub-clone [6].

In general, PTLD and PTSMT can manifest in one patient but are considered unrelated tumour events [3, 4]. In the present case, we do not consider this lesion as a PTLD/PTSMT overlap neoplasm because analyses show PTLD-typical genetic defects, while PTSMT usually show no c-MYC/IGH rearrangements [3]. The differential diagnostic pitfall is the negativity of B and T cell markers, including PAX5, and the positivity for actin and desmin. Therefore, in this rare cases we recommend the evaluation of plasma cell and muscle markers, such as caldesmon, PCR and fluorescence in situ hybridisation, in order to differentiate between PTLD and PTSMT. However, we do not recommend routine testing of mesenchymal markers in PTLD because CD20 negativity is the most important adverse prognostic factor in PTLD and not anaplasia and/or aberrant marker expression itself [7].

While reduced immunosuppression can be applied in both tumour types, diagnostic discrimination remains relevant because different therapy strategies should be initiated. Rituximab therapy will be inefficient in patients with CD20^−^ PTLD, who may instead benefit from standard anti-lymphoma chemotherapy, despite the poorer prognosis in comparison with CD20^+^ PTLD patients [7]. If possible, surgery should be performed in patients with PTSMT because anti-sarcoma chemotherapy and/or radiation are often not effective [3].

In summary, this case shows that aberrant mesenchymal marker expression in CD20^−^ PTLD could be a pitfall, potentially leading to misdiagnosis of a PTSMT.
